# Working Length Determination Using Cone-Beam Computed Tomography, Periapical Radiography and Electronic Apex Locator in Teeth with Apical Periodontitis: A Clinical Study

**DOI:** 10.7508/iej.2016.03.003

**Published:** 2016-05-01

**Authors:** André Luiz Gomide de Morais, Ana Helena Gonçalves de Alencar, Cyntia Rodrigues de Araújo Estrela, Daniel Almeida Decurcio, Carlos Estrela

**Affiliations:** a *Dental School, Federal University of Goiás, Goiânia, GO, Brazil; *; b * Department of Endodontics, Federal University of Goiás, Goiânia, Go, Brazil; *; c * Department of Endodontics, University of Cuiabá, Cuiabá, MT, Brazil*

**Keywords:** Apical Foramen, Cone-Beam Computed Tomography, Dental Radiography, Electronic Apex Locator, Tooth Apex

## Abstract

**Introduction:**

The purpose of this clinical study was to compare the accuracy of working length (WL) determination using cone-beam computed tomography (CBCT), conventional periapical radiographies and electronic apex locator.

**Methods and Materials:**

This study was conducted during root canal treatment of 19 patients with a total of 30 single-rooted teeth diagnosed with apical periodontitis. After taking the initial parallel periapical radiographies, the initial file was advanced into the canal until the WL was detected by the apex locator. Subsequently, the WL was measured and WL radiographies were taken with the file set in the canal. Afterwards, CBCT images were acquired. These three measurements were tabulated and compared and the data were analyzed using the Friedman test. The level of significance was set at 0.05.

**Results:**

The mean values for WL determination by electronic apex locator, periapical radiograph and CBCT images were 22.25, 22.43 and 22.65, respectively which was not statistically significant (*P*>0.05).

**Conclusion:**

Working length determination using CBCT images was precise when compared to radiographic method and electronic apex locator.

## Introduction

The structural complexity of the apical third of the root canals in teeth with apical periodontitis may hamper the endodontic therapy. Elimination of microorganisms harbored within the root canal system, depends on the sequence of operative steps, such as coronal access, root canal preparation and filling [1, 2]. A better understanding of variations in the internal anatomy of root canals and obtaining the working length (WL) where the mechanical preparation and chemical irrigation is limited to, is a challenge for contemporary endodontics [1-3].

The prognosis of endodontic therapy is strongly influenced by apical limit of root canal instrumentation and obturation. In separate meta-analyses by Kojima *et al.* [4] and Schaeffer *et al.* [5], the ideal limit for ending the instrumentation and root canal filling was discussed. It was stated that the apical limit is affected by the pathological status of the pulp and periodontium. A higher success rate was observed when treatment was limited short of the radiographic apex.

Most endodontists prefer a combination of electronic apex locators and radiographic methods to determine the WL [6-8]. Although the paralleling technique reduces the dimensional changes in the final radiographic image, the ideal orientation of the x-ray tube are difficult aspects to standardize. Distorted radiographic images (stretched or shortened) may result from errors in the vertical positioning of the film or from angulation of the radiographic positioner [7, 8]. Changes in the angle between the radiographic film and the tooth have a significant effect on linear measures based on periapical radiographies [9, 10].

Several devices were developed in order to find the most appropriate length for instrumentation and obturation of root canals. The development of new technologies has make endodontic therapy more rational, less stressful for the professionals and more accurate [11-18]. The electronic apex locators reduce the number of required radiographies and minimize the subjectivity involved in radiographic interpretation.

The basis of WL determination by apex locators include electronic measurements of root canal length based on the resistance, low-frequency oscillation, high frequency, capacitance and resistance devices, equipment based on voltage gradient, on two frequencies and impedance difference, on two frequencies and impedance ratio and on multi-frequency impedance. The most current apex locators measure the impedance difference between two frequencies, or the ratio of two electrical impedances. The accuracy of electronic measuring devices has been the subject of numerous studies [5, 11-18]. Its function constitutes to detect an area between the minor and the major foramen, which represents the transition between the pulp and periodontal tissues, point of reference in which endodontic instrumentation and obturation should terminate preferentially [19-21].

New imaging modalities have been included in clinical practice, such as digital radiography, densitometry, computed tomography, magnetic resonance imaging, ultrasound and nuclear techniques [22-28]. These detailed images show the oral structures in a high-resolution and allow the early detection of changes in maxillofacial structures. Cone-beam computed tomography (CBCT) represents an important technology recently introduced to dentistry, with high potential for clinical application and accuracy compared to periapical radiography. Its contribution to the treatment plan, diagnosis, treatment and prognosis of different diseases, besides its value in research are observed [23-28].

CBCT scans can be used to allow more accurate WL measurements, offering the advantage of this preexisting information [29]. The null hypothesis of this study is related to the absence of differences between the measurement of WL obtained by the CBCT images, periapical radiographies and electronic apex locators. Thus, the purpose of this clinical study was to compare the accuracy of WL determined by CBCT with periapical radiographies and electronic apex locators.

## Materials and Methods

This preliminary clinical study evaluated 30 single-rooted teeth (including 13 central incisors, 14 lateral incisors and 3 canines from maxilla) from 19 patients (12 female and 7 male patients with the mean age of 33.8) referred to Dental Urgency Service of the School of Dentistry of Federal University of Goiás, Brazil, from 2009 to 2010. In all patients the diagnosis was asymptomatic apical periodontitis associated with primary infection. 

This study was approved by the Institutional Ethics in Research Committee of Federal University of Goiás, Goiânia, Brazil. All patients were informed of the study and signed an informed consent form.

Initial diagnostic radiographies were taken with parallel technique. Teeth with calcifications of the root canal, internal or external resorption, metallic restorations, root fractures and absence of fully formed root apices, were excluded. All periapical radiographies were obtained using Spectro 70× x-ray machine (Dabi Atlante, Ribeirão Preto, SP, Brazil), with focal tube of 0.8×0.8 mm and Kodak Insight E-films (Eastman Kodak Co, Rochester, NY, USA). All films were processed in an automatic processor.

The root canal treatment of all teeth was performed by an endodontist who was blinded to the results of the CBCT measurements. The access cavities were prepared using #1012 and 2200 diamond burs (KG Sorensen, Agerskov, Denmark). After locating the root canals they were copiously irrigated with 5 mL of 2.5% hypochlorite sodium. The canals were explored using a size 15 stainless steel K-file (Dentsply-Maillefer, Ballaigues, Switzerland). The preparation of the coronal zones of canals was done with # 2 and 3 Gates-Glidden drills (Dentsply-Maillefer, Ballaigues, Switzerland) and #1 and 2 Largo burs (Dentsply-Maillefer, Ballaigues, Switzerland).

Then, by advancing a stainless steel K-file that best suited the root canal patency the WL was determined, using an electronic apex locator (Root ZX apex locator, J. Morita USA, Inc., Irvine, CA, USA). The silicon stopper on the inserted file was then set to an anatomical tooth landmark, the file was retracted and the distance between the stopper and the file tip was measured using a millimeter ruler. After that, the silicon stopper was retreated 1.0 mm and this measurement was noted. 

The radiographic measurement was made by advancing a K-file in the root canal, until its tip was 1.0 mm from the root apex (determined from the measures obtained by the electronic apex locator). Radiography was exposed and if the file tip was not 1.0 mm short from the radiographic apex, the file was repositioned and another radiography was taken to ensure that it was in the right measurement. The distance from the stop to the file tip was noted. 

**Figure 1:**
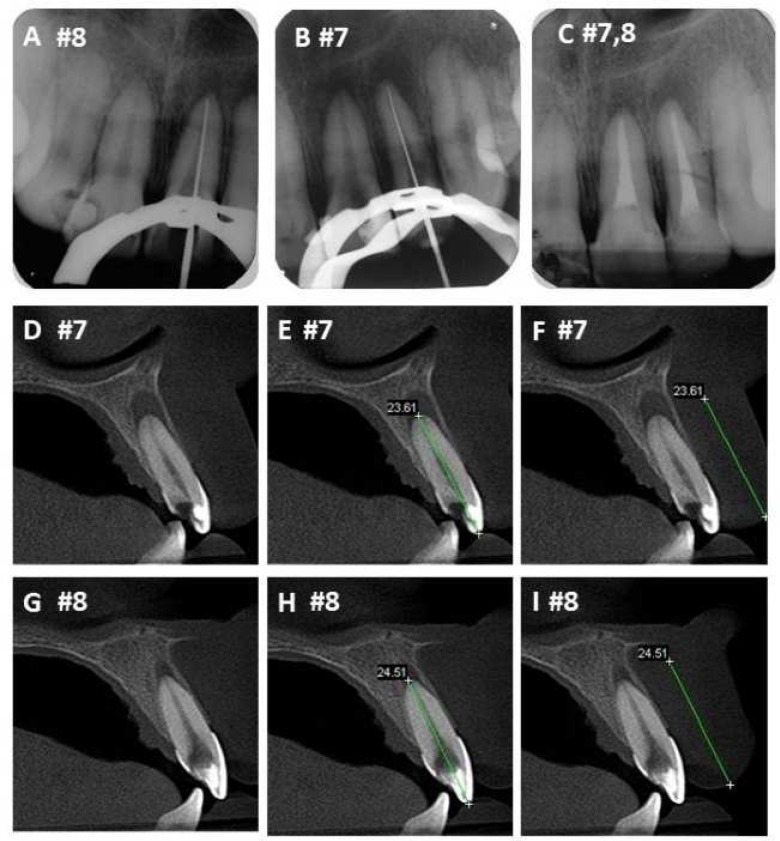
Determination of working length in maxillary central and lateral incisors: *A to C)* periapical radiographies and *D to I)* CBCT images

CBCT images were acquired with i-CAT Cone-Beam 3D imaging system (Imaging Sciences International, Hatfield, PA, USA). The tube voltage was 120 kVp and the tube current was 3.8 mA. Exposure time was 40 sec. Images were examined with the scanner’s proprietary software (Xoranversion 3.1.62; Xoran Technologies, Ann Arbor, MI, USA). The method used to study the root canal WL determination with CBCT was based on delimiting and measuring the distance between anatomical landmarks of the dental crowns and roots. All the measurements on the CBCT images were acquired by a dental radiology specialist, using a proprietary measurement tool supplied with the CBCT scanner (Xoran 3.1.62; Xoran Technologies, Ann Arbor, MI, USA). A specific function of i-CAT software that offers values in millimeters was used to measure tooth images. The measurements were made in the sagittal plane (the reference was the largest measurement extension given by the software). The reference distance used was the maximum width between the incisal edge or cusp tip and the most apical point of the root. The measurement was 1.0 mm short of the root apex (Figure 1).

The Shapiro-Wilk test was used to verify the distribution of aleatory errors around the means (normality). The Friedman test was used to compare the WL measured with CBCT, radiographic imaging or electronic apex locator using SigmaPlot for Windows (Version 12.0, Systat Software Inc., San Jose, CA, USA). The level of significance was set at 0.05.

## Results

The mean values of WL was obtained by using the electronic apex locator, the periapical radiographies and CBCT images are described and are presented in Table 1. The distribution of data was not normal (*P*<0.05). The values obtained by electronic apex locator, periapical radiographies and CBCT images were 22.25, 22.43 and 22.65, respectively. No statistical significant differences were found between the three methods (*P*>0.05).

## Discussion

Different strategies have been used to determine the position of the apical foramen and to thus to measure the WL of root canals [11-19]. The most widely used method is taking periapical radiographies. However, although the accepted place for apical constriction is between 0.5 to 1.0 mm from the radiographic apex, there are variations in the relationship from that point of reference which result in errors of instrumentation, and that obviously influence the position of endodontic filling. References of anatomical structures visualized on radiographies can be showed as hidden [19-21, 30].

Electronic devices to detect the end of the root canal represent important innovations in endodontic treatment [11, 13-18, 31, 32]. The functionality of these equipments are based on the fact that the electrical conductivity of the tissues surrounding the root apex is greater than the conductivity inside root canal system being the channel dry or filled by non-conductive fluid [11-18, 31, 32].

The present study considered the recommendations suggested by the American Association of Endodontists and the American Academy of Oral and Maxillofacial Radiology [33] for the use of CBCT in endodontic treatment, such as the diagnosis of dental periapical pathosis in patients who present with contradictory or nonspecific clinical signs and symptoms, who have poorly localized symptoms associated with an endodontically untreated tooth.

The results of the current study are in accordance with previous reports which analyzed the potential of CBCT in root-length measurements [10, 30, 34, 35]. Janner *et al.* [34] in a pilot investigation evaluated the utility and precision of already existing CBCT scans in measuring the endodontic WL, and compared it with standard clinical procedures. A strong correlation was found between the endodontic WL measured in the CBCT images and the electronic apex locator measurements. Sherrard *et al.* [10] evaluated the accuracy and reliability of tooth-length and root-length measurements derived from CBCT volumetric data made from 7 fresh porcine heads. CBCT scans are at least as accurate and reliable as periapical radiographies for determining root length. Differences between the length of root canal obturation detected by periapical radiographies and CBCT images were showed by Moura *et al.* [30]. Periapical radiographies showed that root canal obturations were 1-2 mm short of the apex in 88%, 89.3%, and 95% of the anterior teeth, premolars and molars, respectively. CBCT images showed that root obturation had the same length in 70%, 73.7%, and 79% of anterior teeth, premolars, and molars, respectively. The frequency of apical periodontitis was significantly greater in molars compared to the other teeth, regardless of the diagnostic method.

Despite the well-known benefits and limitations of periapical radiographies [22-26, 32], and to represent the common devices for regulating root canal WL, several professionals have chosen the electronic apex locator [32, 36, 37]. Shabahang *et al.* [36] verified that Root ZX was able to locate the foramen with a clinical accuracy rate of 96.2%. Ravanshad *et al.* [37] compared the effect of WL determination using electronic apex locator or radiography on the length adequacy of final WL as well as the final obturation in 84 patients. The endodontic treatment using the electronic apex locator is quite comparable, if not superior, to radiographic length measurement regarding the rates of acceptable and short cases.

The influence of the apical limits determination for root canal preparation and obturation on outcome of root canal treatment, and the available resources to evaluate the root canal WL correctly have been the subject of constant discussions [4, 5, 19, 21, 30]. The criteria for successful root canal treatment should be reviewed based on the results achieved with new technologies such as CBCT [28, 29, 38]. Periapical radiography has been employed to assess the WL as well as the outcome of root canal treatment. 

Based on the possibility that CBCT images may be instructed for teeth with apical periodontitis as a diagnostic aid, it may favor tooth length achievement. Thus, it is possible to confirm this length by using electronic apex locators avoiding a new radiographic exposure.

**Table 1:** Comparison between working length medians obtained using electronic apex locator, periapical radiography or CBCT images

**Apex locator**	**Radiography**	**CBCT image**	***P-*** **value**
22.25	22.43	22.65	0.053

## Conclusion

The determination of the working length of root canal using CBCT images was precise when compared to radiographic method and electronic apex locator.
